# Supplementary material for: Mutation of Leaf Senescence 1 Encoding a C2H2 Zinc Finger Protein Induces ROS Accumulation and Accelerates Leaf Senescence in Rice
Source: Int J Mol Sci. 2022 Nov 21;23(22):14464. doi: 10.3390/ijms232214464 (PMC9696409; doi:10.3390/ijms232214464)
Supplement: Supplementary file 1 [file ijms-23-14464-s001.zip › Supplementary Material.pdf]

Supplementary Material

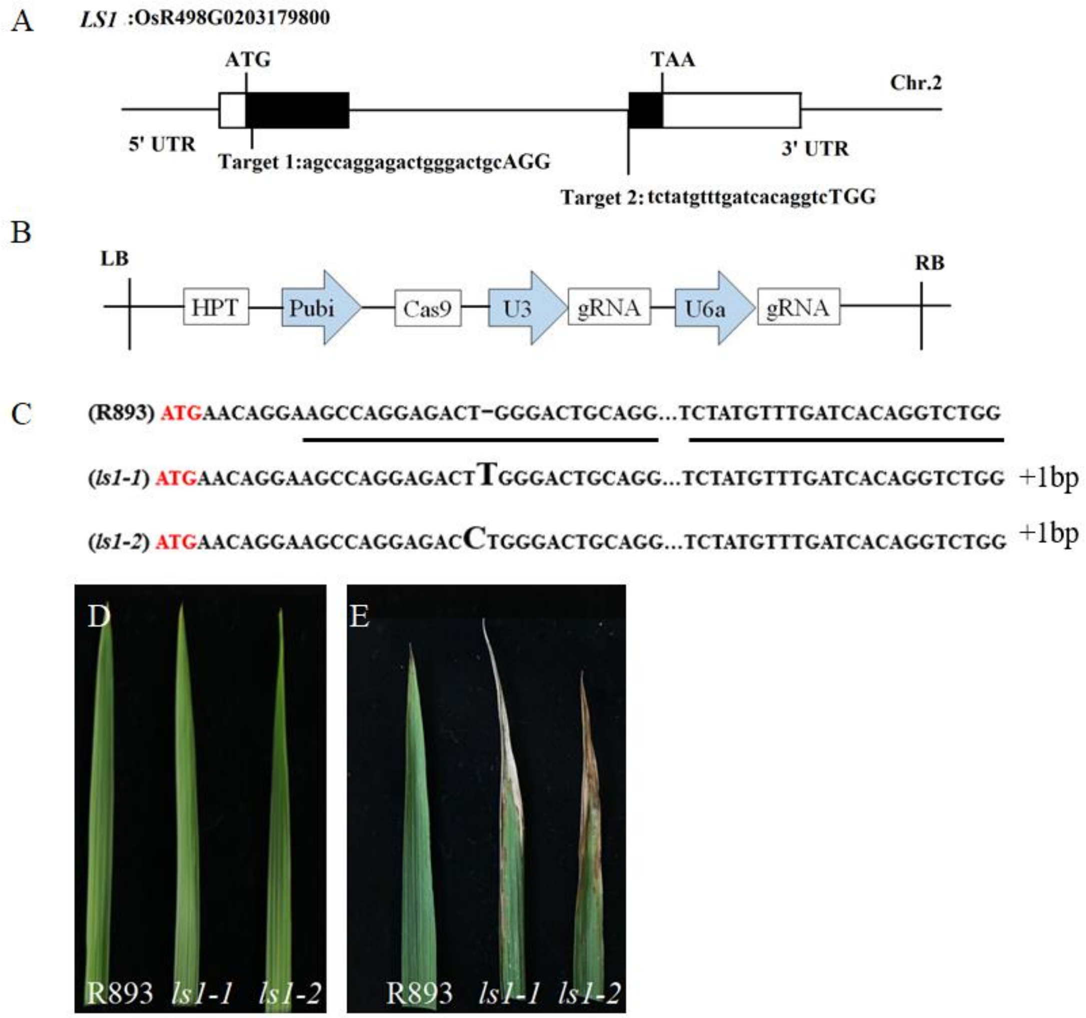

Figure S1. Target sites of the CRISPR/Cas9-*LS1*-T1, T2 vector and phenotypic of the *ls1* plants and wild type R893. **(A)** Schematic of the *LS1* gene structure and target site. Coding sequence (CDS), introns and untranslated region (UTR) are indicated with black rectangles, black lines and white rectangles, respectively. Both the translation initiation codon (ATG) and the termination codon (TAG) are shown. The target site nucleotides are shown in lowercase. **(B)** A schematic presentation of the T-DNA structure in the CRISPR/Cas9-mediated genome editing construct. The expression of Cas9 is driven by the maize ubiquitin promoter (Pubi); the expression of the sgRNA frame is driven by the rice U3 small nuclear RNA promoter; the expression of hygromycin (HPT) is driven by CaMV35S promoter; LB and RB, left border and right border, respectively**. (C)** Mutation sites of *LS1* gene in *ls1-1* and *ls1*-2 mutants. The target site nucleotides are indicated using black lines. The translation initiation codon (ATG) is highlighted in red. The numbers on the right indicate the type of mutation and the number of nucleotides involved. "+" indicate the insertion. **(D,E)** Leaf phenotypic comparison of wild type R893 (WT), *ls1-1*, *ls1-2* mutant. Leaves were taken from plants grown in the paddy fifield at the seedling stage (**D**) and heading stage (**E**), respectively.

Table S1. Primers of vector construction used in this study

Primers	Forward primer sequence (5′to3′)	Reverse primer sequence (5′to3′)
T1	GGCAGCCAGGAGACTGGGACTGC	AAACGCAGTCCCAGTCTCCTGGCT
T2	GCCGTCTATGTTTGATCACAGGTC	AAACGACCTGTGATCAAACATAGA
F-U/ R- gRNA	CTCCGTTTTACCTGTGGAATCG	CGGAGGAAAATTCCATCCAC
F-T1/ R- T2	GGCAGCCAGGAGACTGGGACTGC	AAACGACCTGTGATCAAACATAGA
F-B1	TTCAGAGGTCTCTCTCGCACTGGAATCGGCAGCAAAGG	
B2-R	AGCGTGGGTCTCGTCAGGGTCCATCCACTCCAAGCTC	
F-B2	TTCAGAGGTCTCTCTGACACTGGAATCGGCAGCAAAGG	
R-BL	AGCGTGGGTCTCTCGACCGGGTCCATCCACTCCAAGCTC	
LS1-F/ LS1-R	AGCTCACCAACACAGCAG	TTCAAAAGTCCGTAACTACCA
LS1-GFP-F/R	ATGAACAGGAAGCCAGGAGAC	TTACACCTCAGTGCCGGAGTC

Table S2. Primers of qPCR used in this study

Gene	Forward primer sequence (5′-3′)	Reverse primer sequence (5′-3′)
OsActin	ATCACTGCCTTGGCTCCTA	CATCTGCTGGAATGTGCTG
SGR	AGGGGTGGTACAACAAGCTG	GCTCCTTGCGGAAGATGTAG
OsI85	GAGCAACGGCGTGGAGA	GCGGCGGTAGAGGAGATG
SAG12-2	ACTGGGGTGAGAAGGGCTACA	CCTCGTCATCATCATCTTCG
OsI57	GCACGGAGGCGAACGA	CTCCCAGCCTGCAAAGTTCATG
OsNAP	CAAGAAGCCGAACGGTTC	GTTAGAGTGGAGCAGCAT
OsWRKY23	TCCAGTTCCTCTCCCAGTTCTAA	CACATTGTTCTCCTTTTCTTCCC
AOX1a	CTTCGCATCGGACATCCATTA	TCCTCGGCAGTAGACAAACATC
AOX1b	CCTGCTCAGTTCATCACCATCA	GCATAAAACGGAGTGACAATAGC
APX1	AGGTGCCACAAGGAAAGATCTGGT	TCAGCAGGGCTTTGTCACTAGGAA
APX2	TGGGAAGATGCCACAAGGAGAGAT	TCCGCAGCATATTTCTCCACCAGT
APX8	GAGCACACACACTTGGAAGATCAA	TACGATGAGCTTCAGCGTAGTC
SODB	TCCGCCGTATAAACTTGATGCCCT	TGGGTTGCCGTTGTTGTATGCTTC
SODA1	ATCTGGATGGGTGTGGCTAGCTTT	AGTACGCATGCTCCCAGACATCAA
CATA	CAACCGCAACGTCGACAACTTCTT	TTCACCGGCAGCATCAGGTAGTTT
CATB	GCTTGCTTTCTGCCCAGCGATAAT	AAATAGTTTGGGCCAAGACGGTGC
LS1	AGGCTTTGGCACTGGCTCTG	CACTGTTGACGGCAGCATCG

Table S3. Sequence of LS1 and its homologs

Accession number	Sequence
LS1	MNRKPGDWDCRACQHLNFSRRDLCQRCGEPRGAADRGSGGGGDYANFGGR GGSSFGGGFGTGSDVRPGDWYCNCGAHNFASRSSCFKCAAFKDDAAVNSGG AGAFDGGDMSRSRGYGFGSGAARASRPGWKSGDWICTRSGCNEHNFASRME CFRCNAPRDSGTEV
KAG8070361.1	MNRKPGDWDCRACQHLNFSRRDLCQRCGEPRGAVDRGSGGSDYANFGGRGG GSSFGGGFGTGSDVRPGDWYCSCGAHNFASRSNCFKCAAFKDEAAVNSGGAA

	GFDGDMSRSRGYGFSTGAARASRPGWKSGDWICTRSGCNEHNFASRMECFRC<br>NAPRDSGTEV
KAG0532253.1	MNRKPGDWDCRSCQHLNFSRRDICQRCSEPRGVADRGSGGGGDYANFGGRG<br>GSSFGGGFGAGSDVRPGDWYCSCGAHNFASRSSCFKCSAYKEEAAVNSGAGGF<br>DSDMSRSRGYGFGSGAARTNRPGWKSGDWICTRSGCNEHNFASRMECFRCNA<br>PRDSGTEA
CAD6251478.1	MNRKPGDWDCRACQHLNFSRRDICQRCSEPRGVADRGSGGGGDYANFGGRG<br>GSSFGGGFGAGSDVRPGDWYCSCGAHNFASRSSCFKCSAYKEEAAVNSGAGGF<br>DSDMSRSRGYGSGSGAARTNRPGWKSGDWICTRSGCNEHNFASRMECFRCNA<br>PRDSGTEA
XP_039788691.1	MNRKPGDWDCRACQHLNFSRRDLCQRCGEPRGAADRGSGGGGDYANFGGR<br>GGSSFGGGFGAGSDVRPGDWLCSCGAHNFASRSNCFKCSAFKEEAAVNSGAG<br>GFDGDMSRSRYGFGGGAARTNRPGWKSGDWICTRSGCNEHNFASRMECFRC<br>NAPRDSGTEV
XP_004951522.1	MNRKPGDWDCRACQHLNFSRRDLCQRCGEPRGAADRGSGGGGDYANFGGR<br>GGSSFGGGFGAGSDVRPGDWLCNCGAHNFASRSNCFKCSAFKEEAAVNSGAG<br>GFDGDMSRSRYSFGGGAARTNRPGWKSGDWICTRSGCNEHNFASRMECFRCN<br>APRDSGTEV
OEL25621.1	MNRKPGDWDCRACQHLNFSRRDLCQRCGEPRGAADRGSGGGGDYANFGGR<br>GGSSFGGGFGAGSDVRPGDWLCSCGAHNFASRSNCFKCSAFKEEAAVNSGAG<br>GFDGDMSRSRYGFGGGAARTNRPGWKSGDWICTRSGCNEHNFASRMECFRC<br>NAPRDSGSAAMTYDNYNL
XP_037453455.1	MNRKPGDWDCRSCQHLNFSRRDLCQRCGEPRSAADRGSVGGALGGDYANFG<br>GRGVGGSSFGTGFGAGSDVRPGDWYCTCGAHNFASRSSCFKCAAFKEEAAVN<br>GGAGGFDGDMSRSRGFGFGAVGGMGAGAAGGRASRPGWKSGDWICTRSGC<br>NEHNFASRQECFRCNAPRDSGTEV
NP_001132718.1	MNRKPGDWDCRACQHLNFSRRDACQRCSEPRGVGDRSGGGGDLGGRGGSSF<br>GGGFGAGSDVRPGDWYCSCGAHNFASRSNCFRCSAYKEEAAGAFDSDMSRSR<br>GYAGFGSGAAARTNRPGWKSGDWICTRSGCNEHNFASRMECFRCNAPRDSGS<br>AATTTYENYLH
KAH1252849.1	MSRPGDWNCRTCNHLNFQRRESCQRCGEPRSGGGGDYGGGFGGGRGSSSFGF<br>TTGPDVRPGDWYCTVGNCGAHNFASRSSCFKCGAPKEDSSAGPFDADMPRMR<br>PYGFGGGSSARPGWKSGDWICTRSGCNEHNFANRMECYRCNAPRDSSSARFA<br>V
MBA0773876.1	MSRPGDWNCRSCQHLNFQRRDSCQRCGEPRPGGGDRVGDYGSFGGRGGSSFG<br>FTGPDVRPGDWYCTVGNCGAHNFASRSSCFKCGAAKDESSGGFESDIPRMRGY<br>GFSTGSSSRSNWKSGDWICARSGCNEHNFASRMECFRCNAPRDSTHKSSY
NP_001326186.1	MSRPGDWNCRSCSHLNFQRRDSCQRCGDSRSGPGGVGGLDFGNFGGRAMSA<br>FGFTTGSDVRPGDWYCTVGNCGTHNFASRSTCFKCGTFKDETGAGGGGGGIG<br>GPAMFDADIMRSRVPGNGGRSSWKSGDWICTRIGCNEHNFASRMECFRCNAP<br>RDFSN<br>RTSF
