# Supplementary figures and images for: Mutation of Leaf Senescence 1 Encoding a C2H2 Zinc Finger Protein Induces ROS Accumulation and Accelerates Leaf Senescence in Rice
Source: Int J Mol Sci. 2022 Nov 21;23(22):14464. doi: 10.3390/ijms232214464 (PMC9696409; doi:10.3390/ijms232214464)

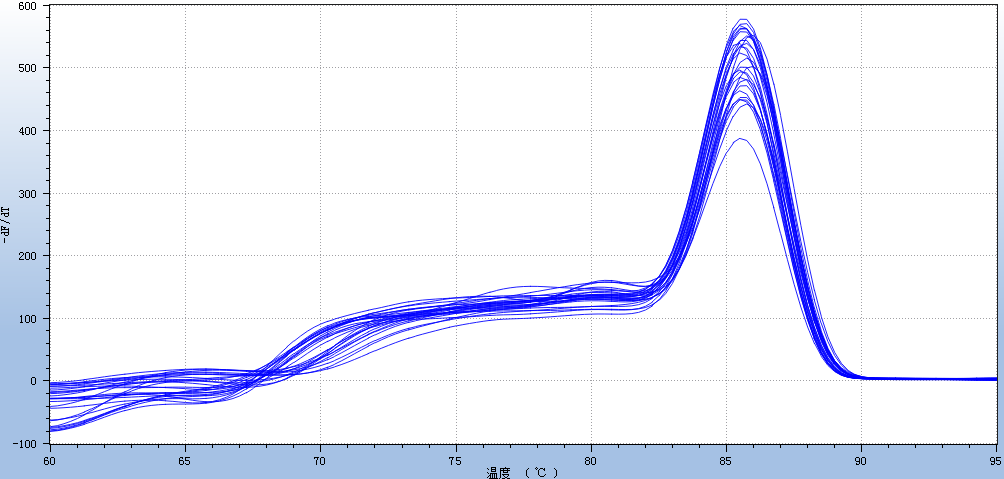

Supplement: Supplementary file 1 [file ijms-23-14464-s001.zip › Melt curve/Actin and LS1/Actin.png]

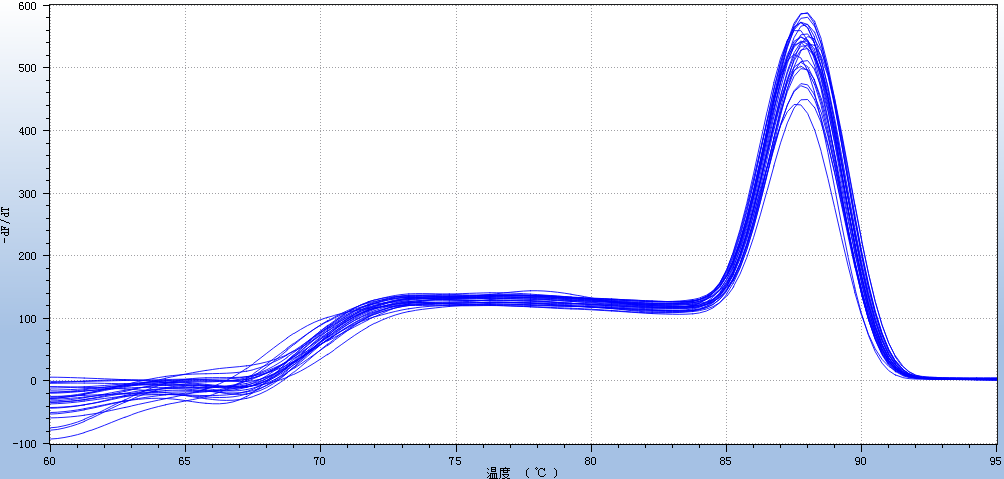

Supplement: Supplementary file 1 [file ijms-23-14464-s001.zip › Melt curve/Actin and LS1/LS1.png]

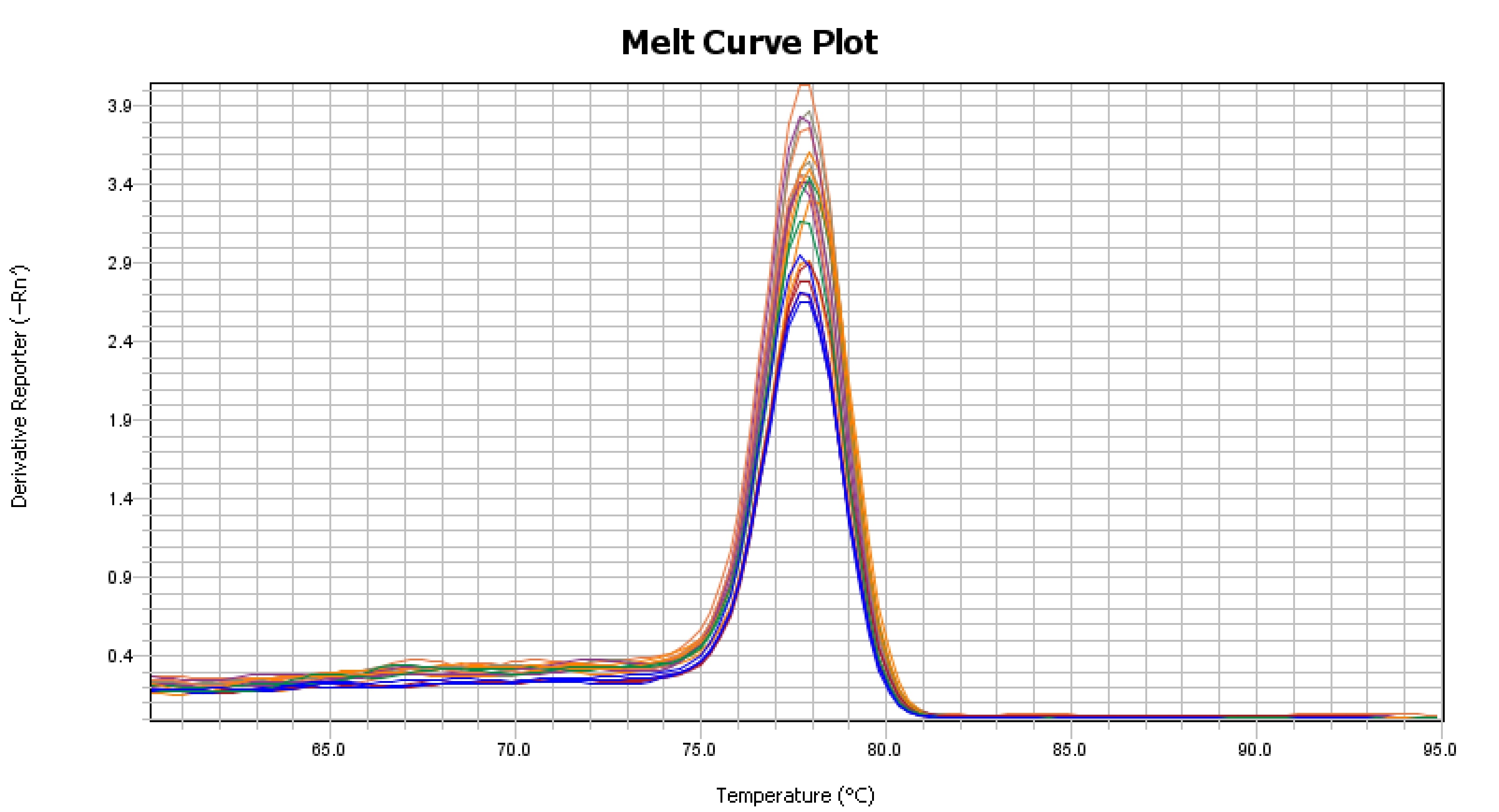

Supplement: Supplementary file 1 [file ijms-23-14464-s001.zip › Melt curve/Senescence associated genes/AOX1a.jpg]

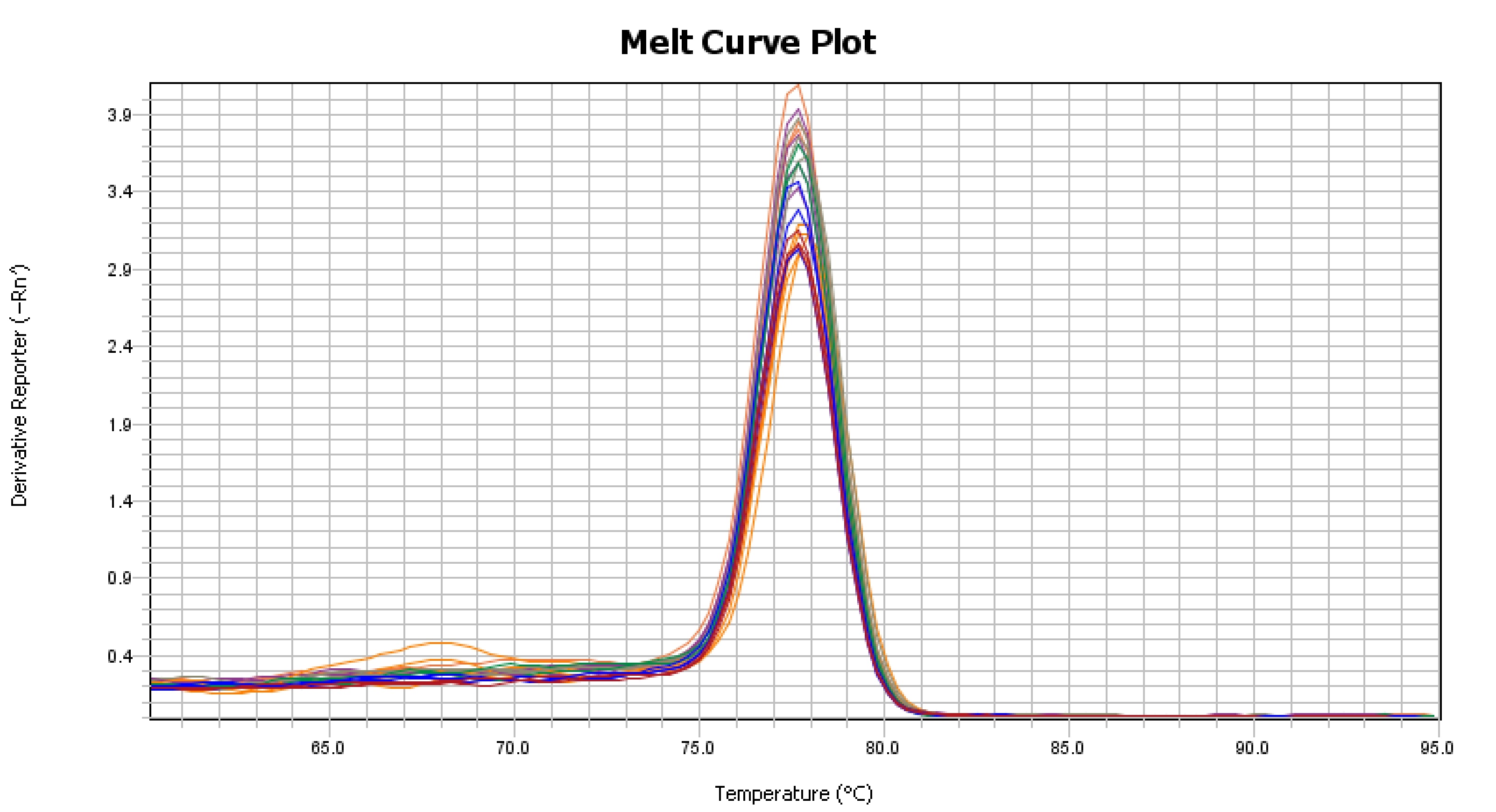

Supplement: Supplementary file 1 [file ijms-23-14464-s001.zip › Melt curve/Senescence associated genes/AOX1b.jpg]

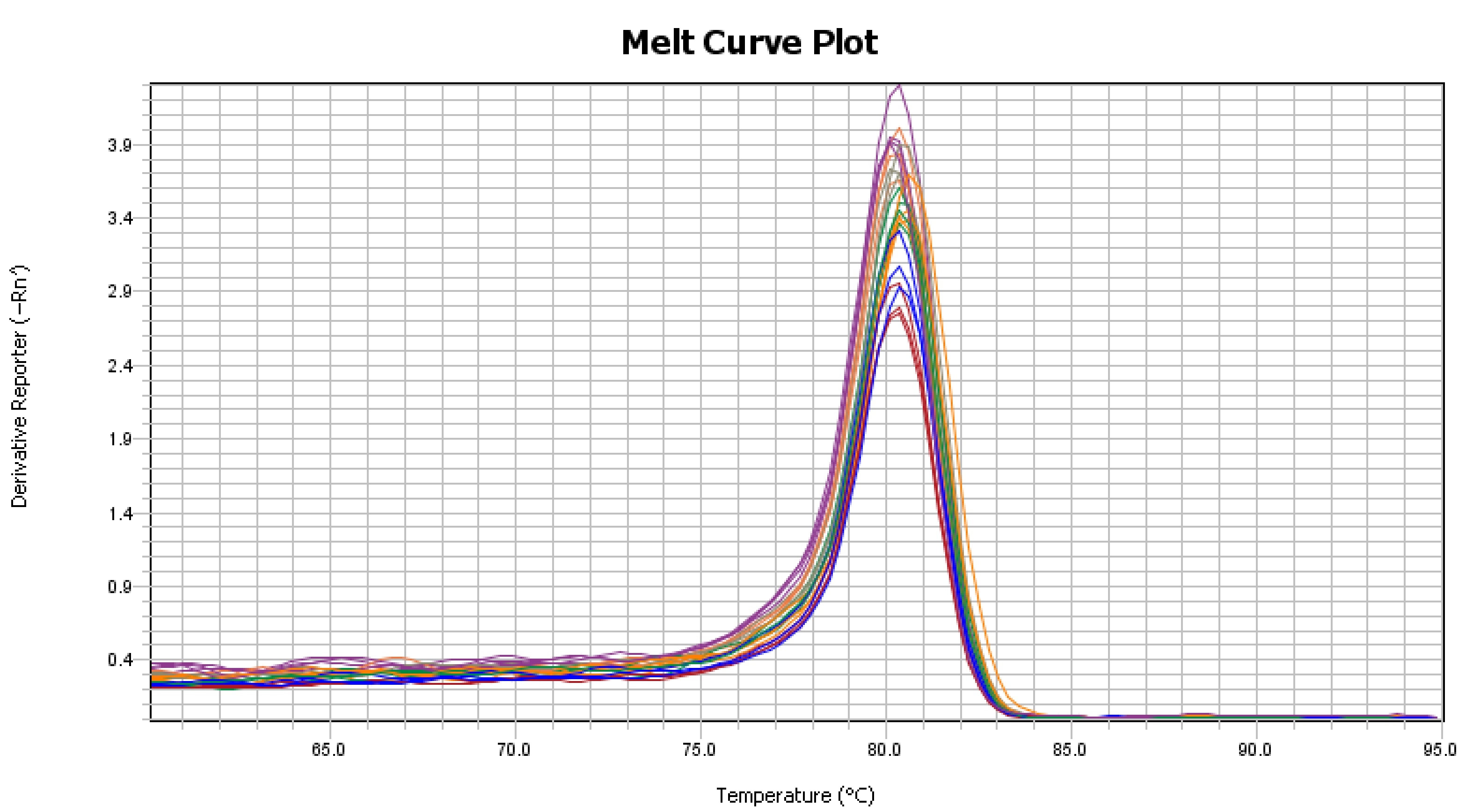

Supplement: Supplementary file 1 [file ijms-23-14464-s001.zip › Melt curve/Senescence associated genes/APX1.jpg]

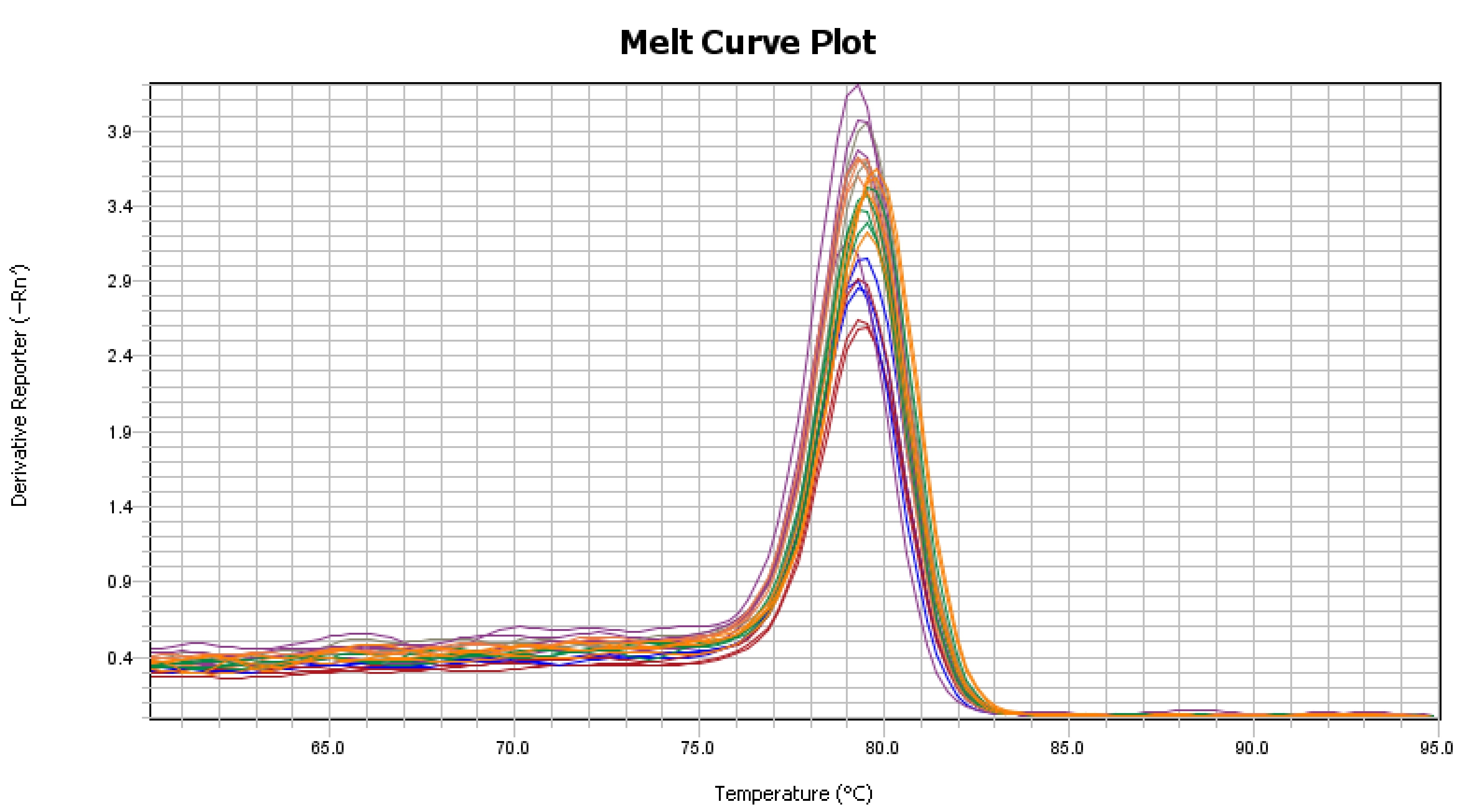

Supplement: Supplementary file 1 [file ijms-23-14464-s001.zip › Melt curve/Senescence associated genes/APX2.jpg]

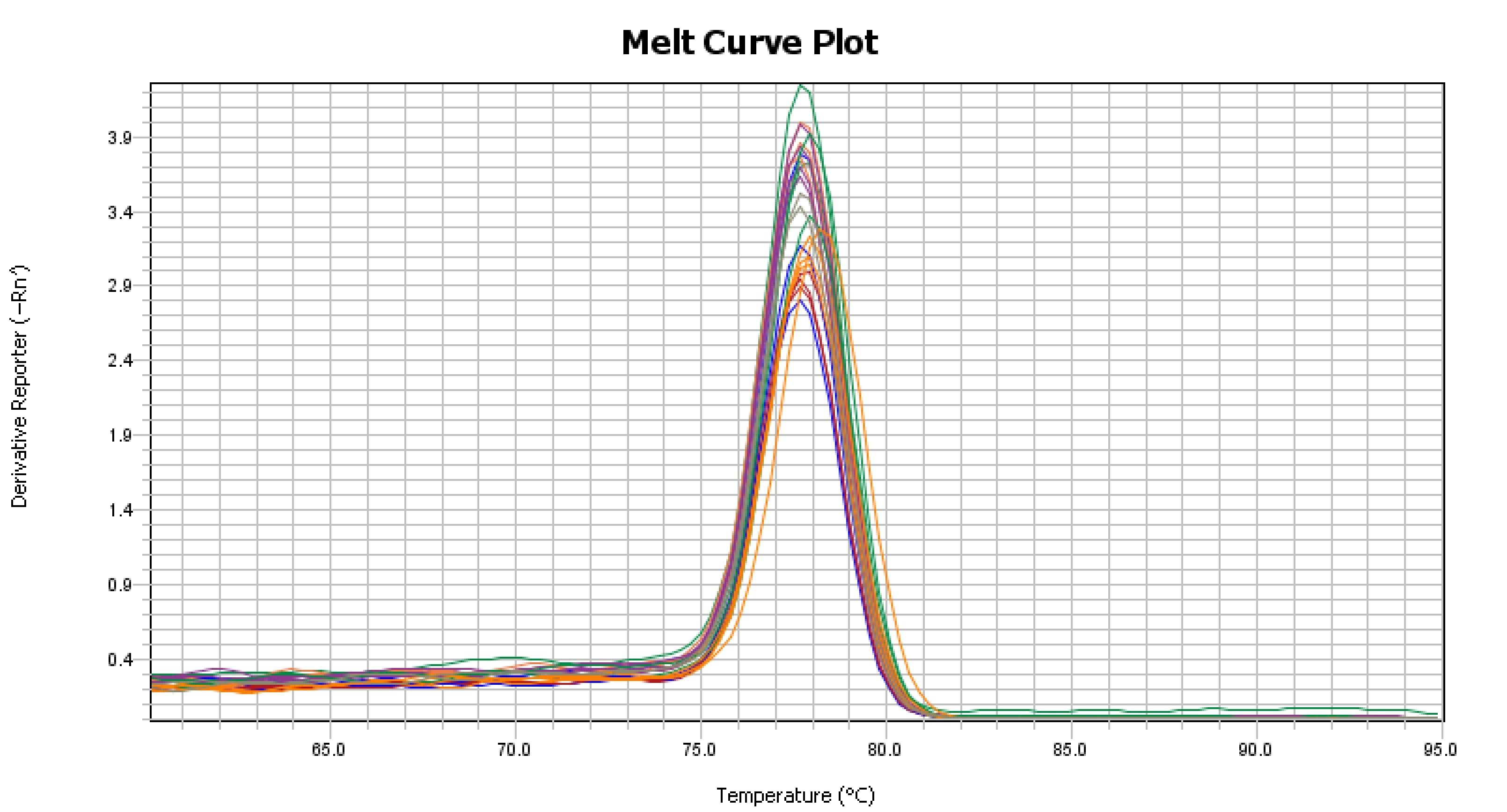

Supplement: Supplementary file 1 [file ijms-23-14464-s001.zip › Melt curve/Senescence associated genes/APX8.jpg]

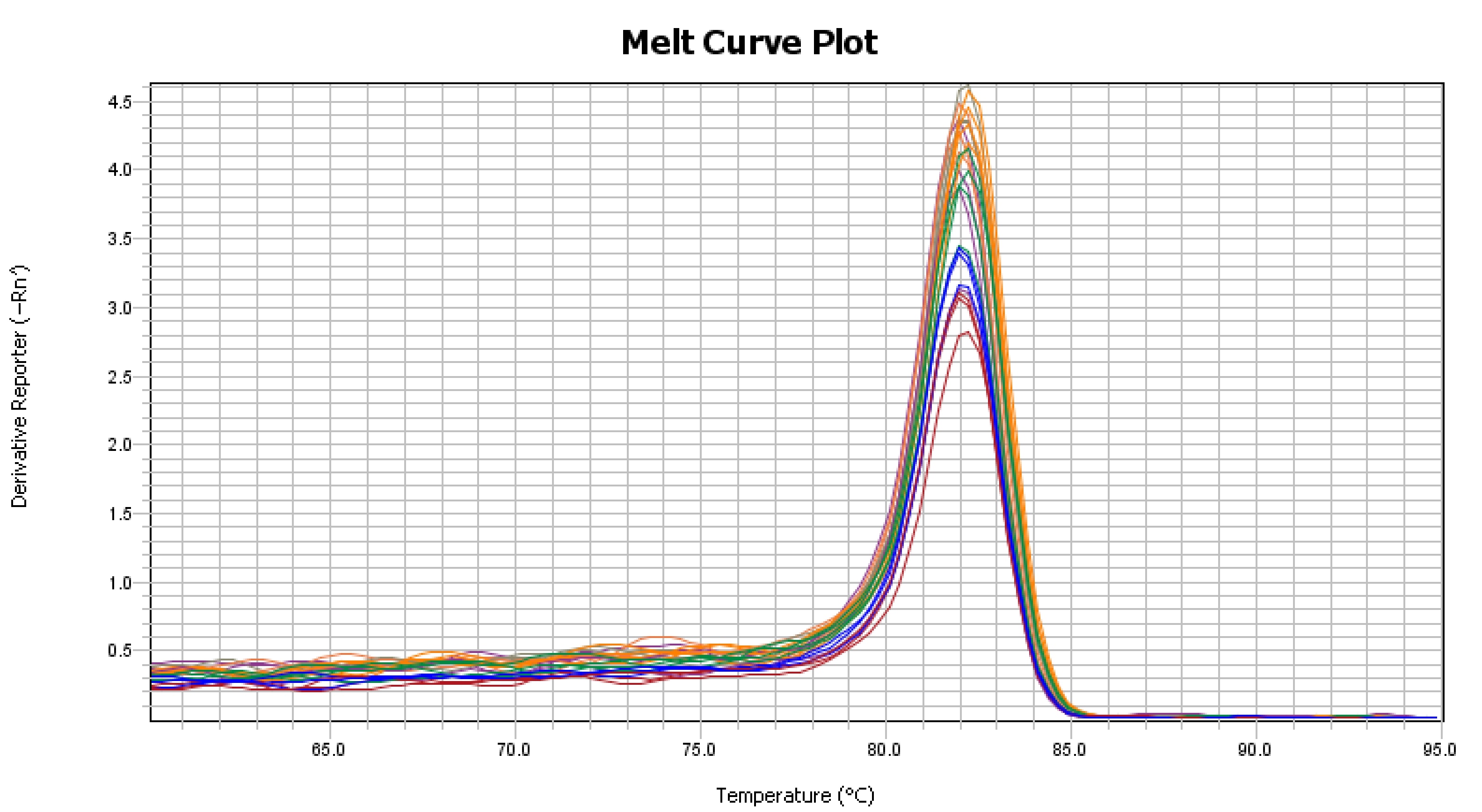

Supplement: Supplementary file 1 [file ijms-23-14464-s001.zip › Melt curve/Senescence associated genes/CATA.jpg]

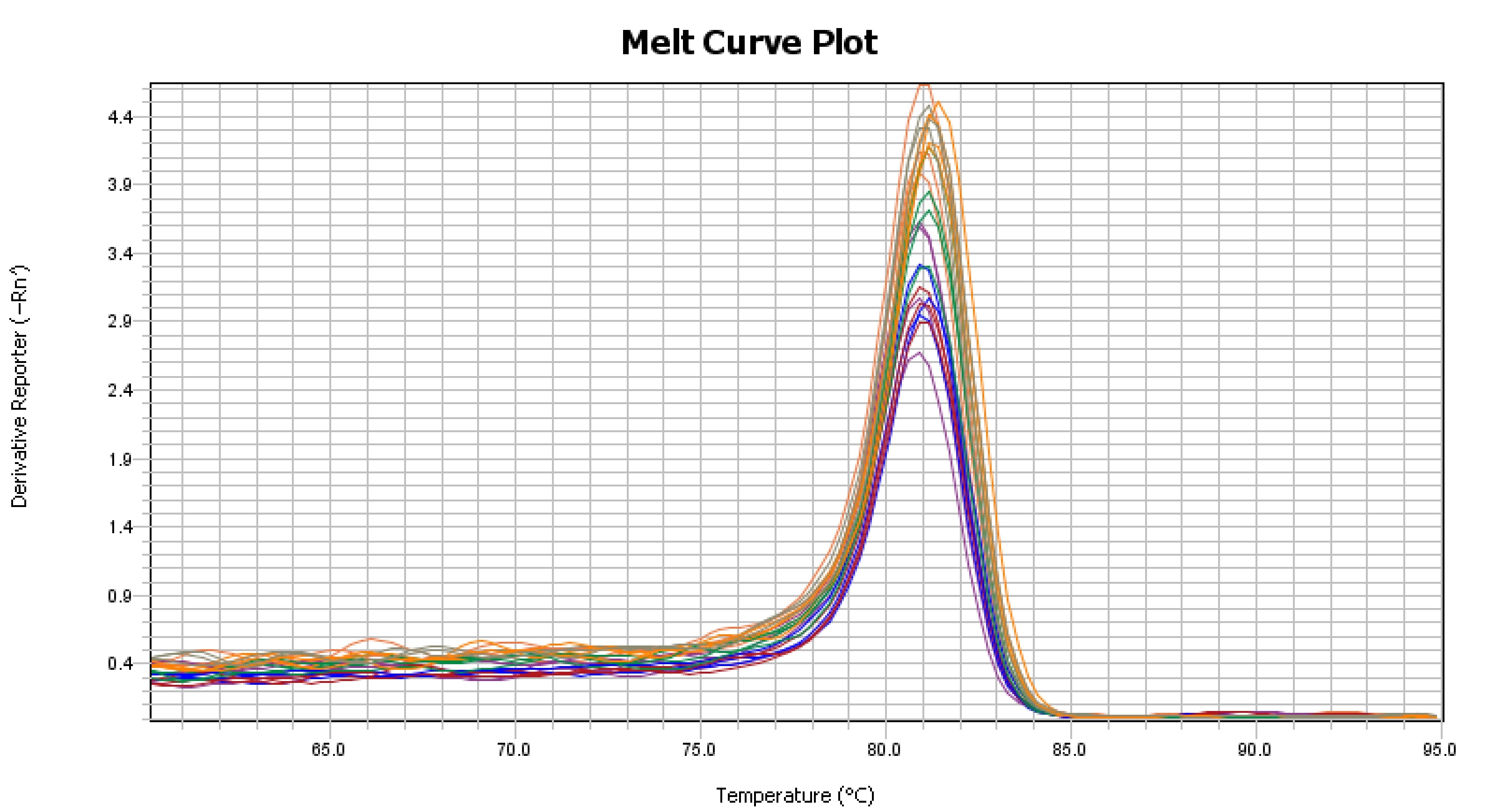

Supplement: Supplementary file 1 [file ijms-23-14464-s001.zip › Melt curve/Senescence associated genes/CATB.jpg]

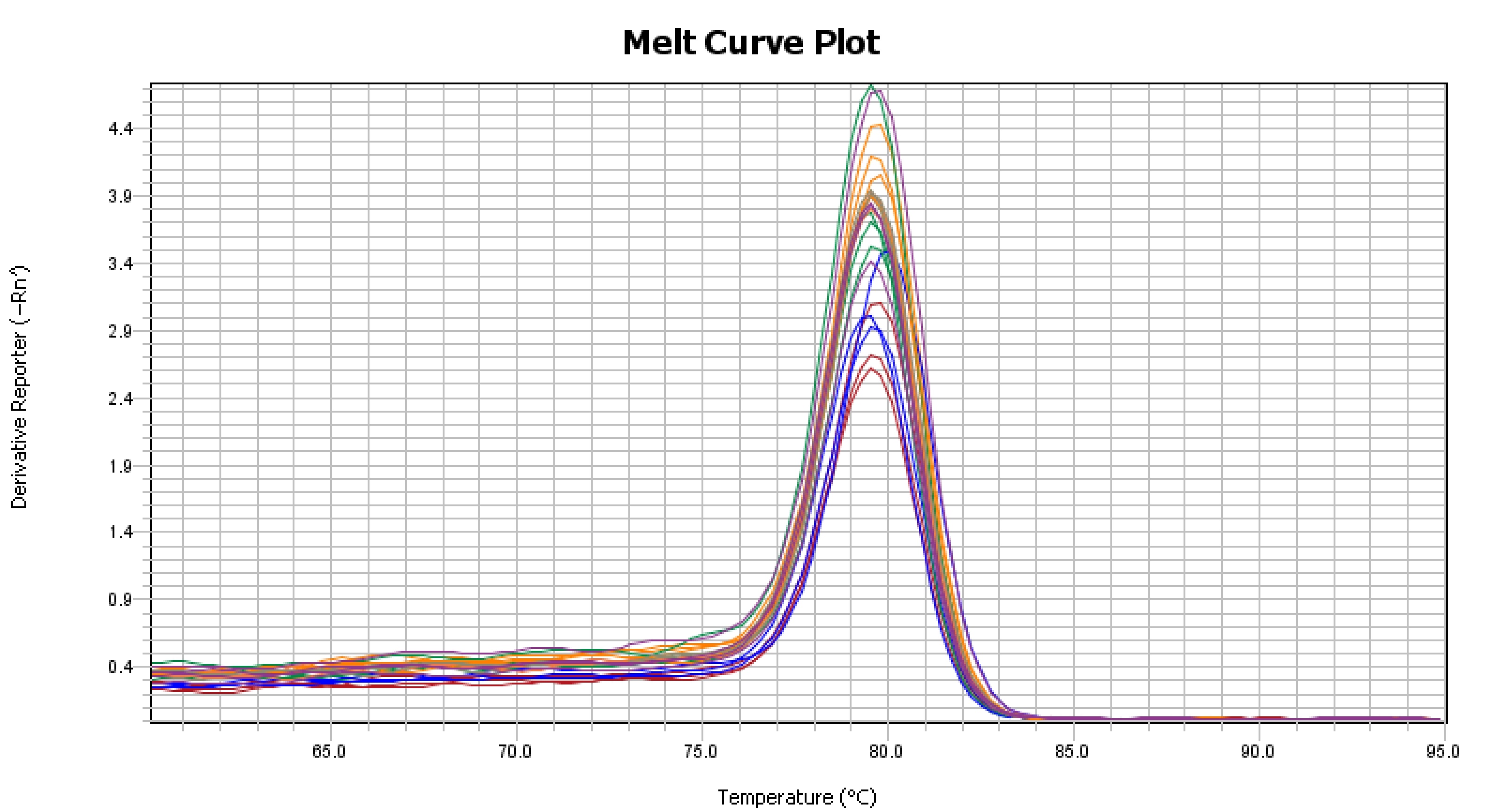

Supplement: Supplementary file 1 [file ijms-23-14464-s001.zip › Melt curve/Senescence associated genes/OsI57.jpg]

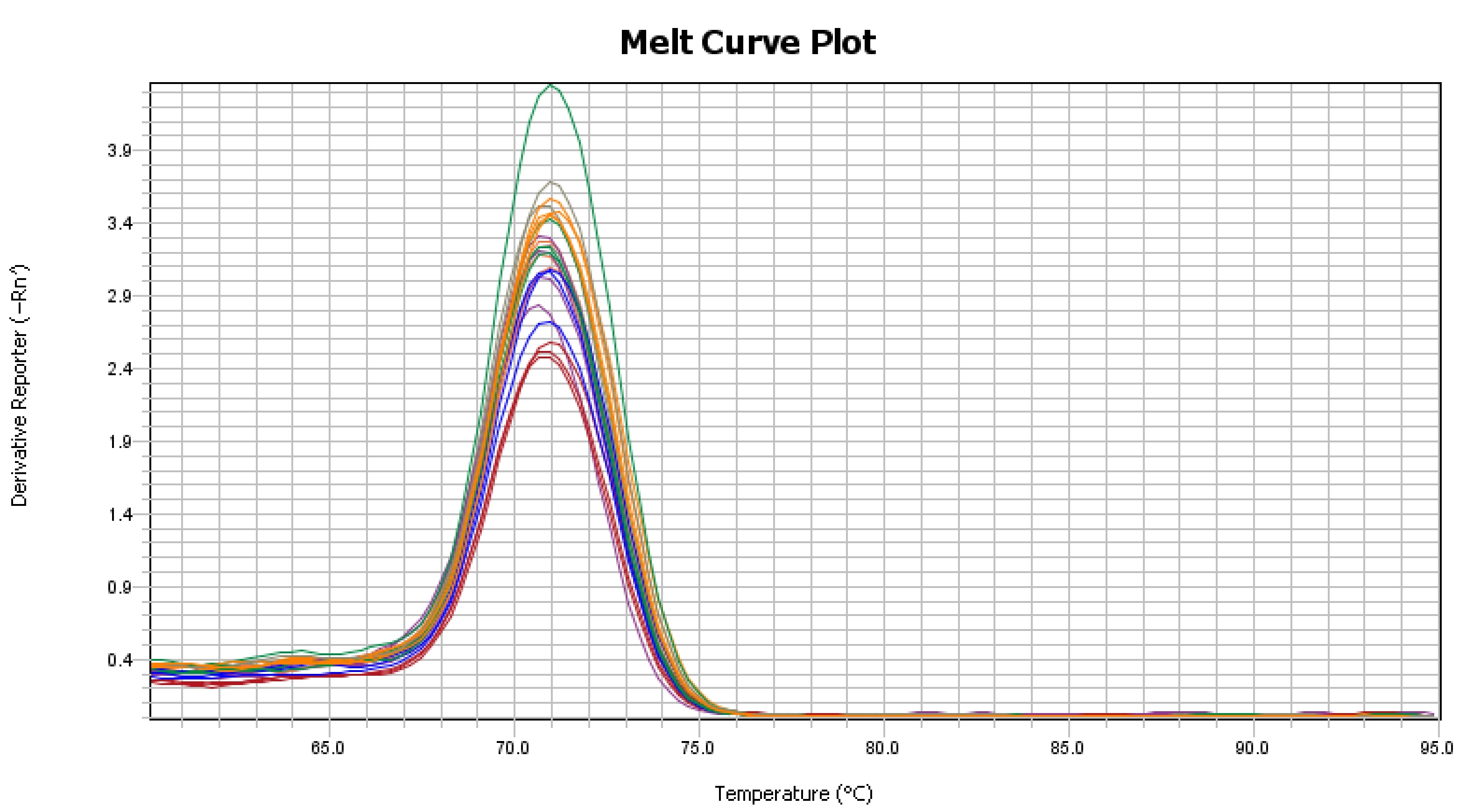

Supplement: Supplementary file 1 [file ijms-23-14464-s001.zip › Melt curve/Senescence associated genes/OsI85.jpg]

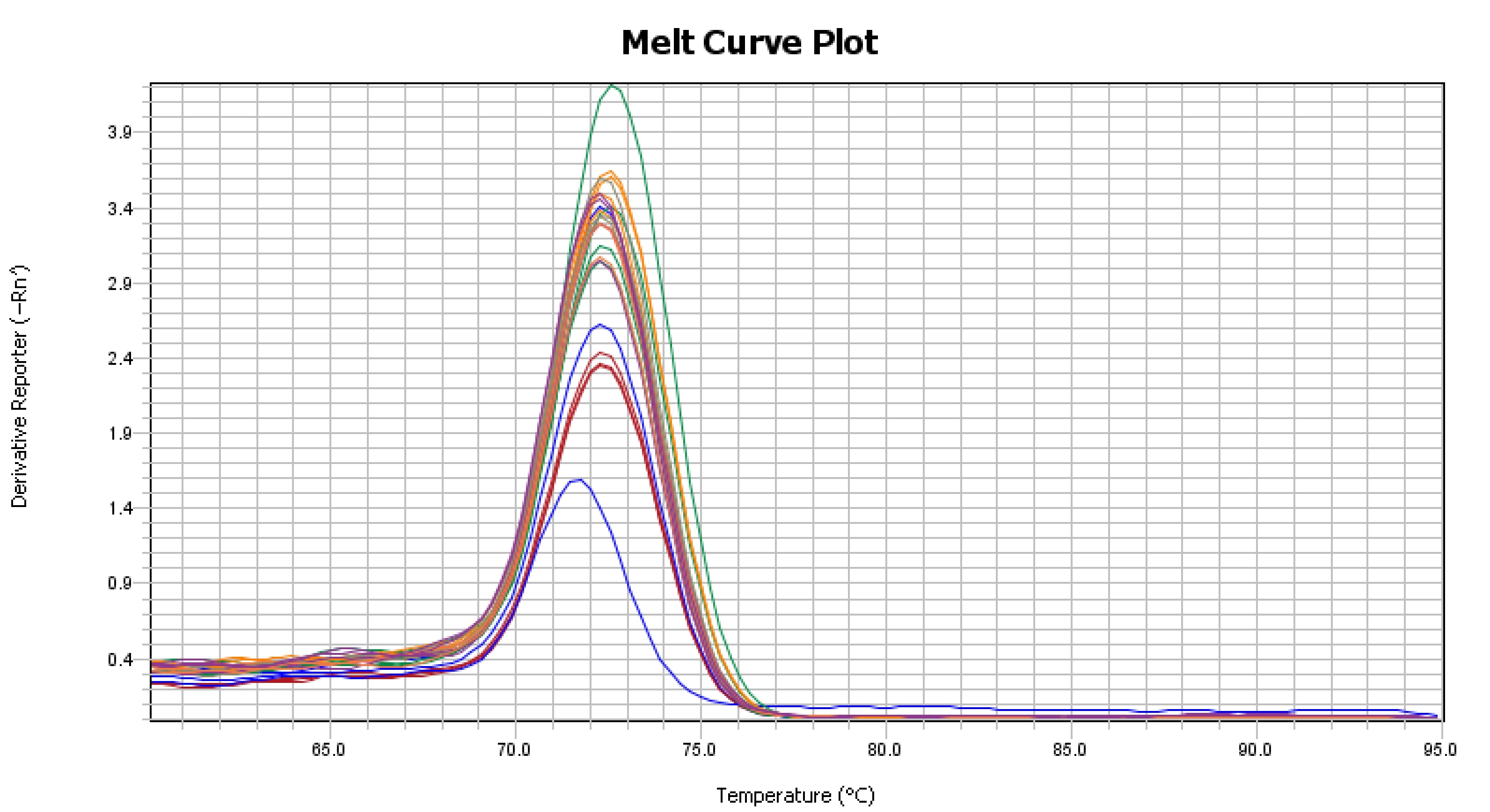

Supplement: Supplementary file 1 [file ijms-23-14464-s001.zip › Melt curve/Senescence associated genes/OsNAP.jpg]

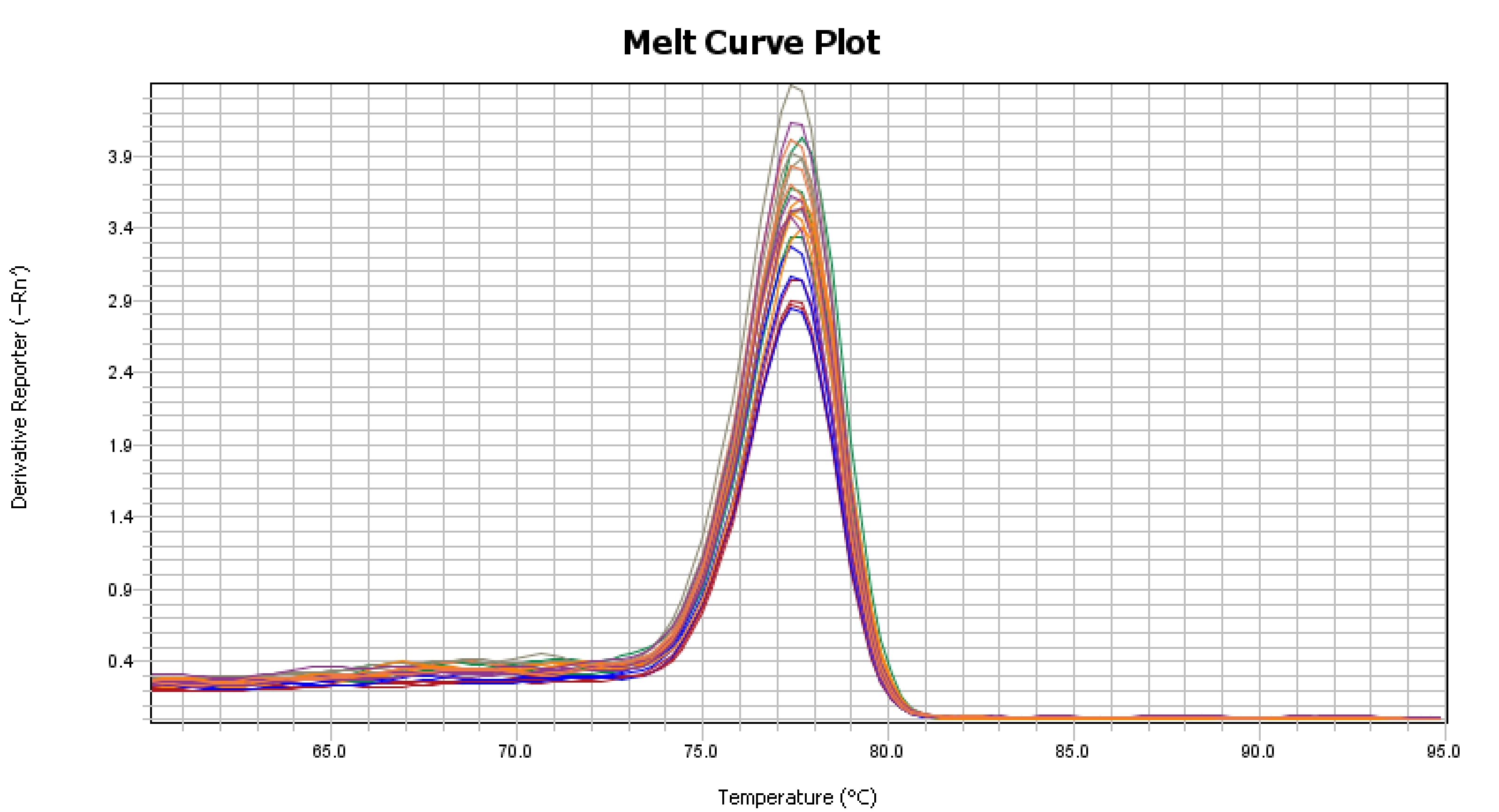

Supplement: Supplementary file 1 [file ijms-23-14464-s001.zip › Melt curve/Senescence associated genes/OsWRKY23.jpg]

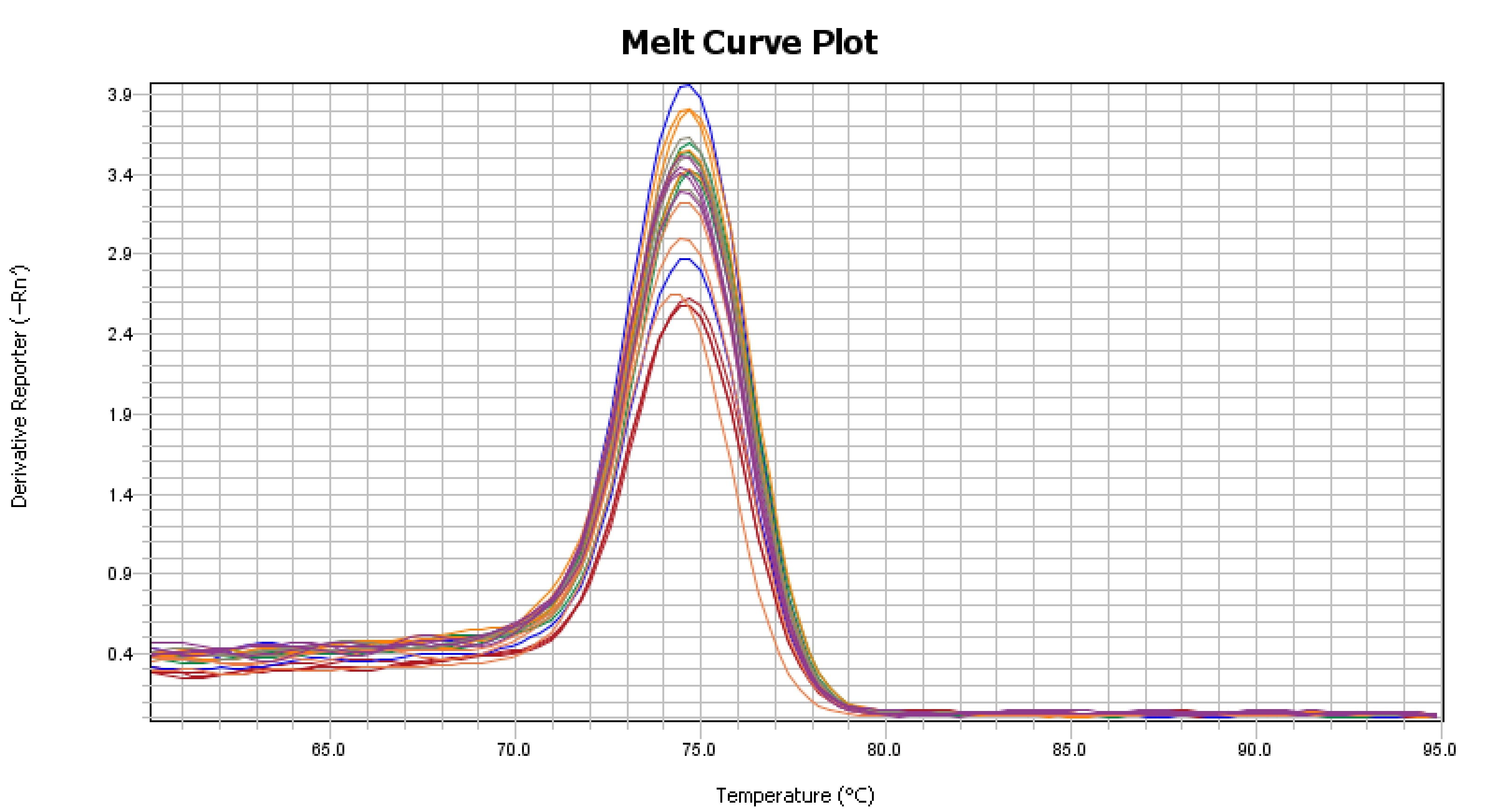

Supplement: Supplementary file 1 [file ijms-23-14464-s001.zip › Melt curve/Senescence associated genes/SAG12-2.jpg]

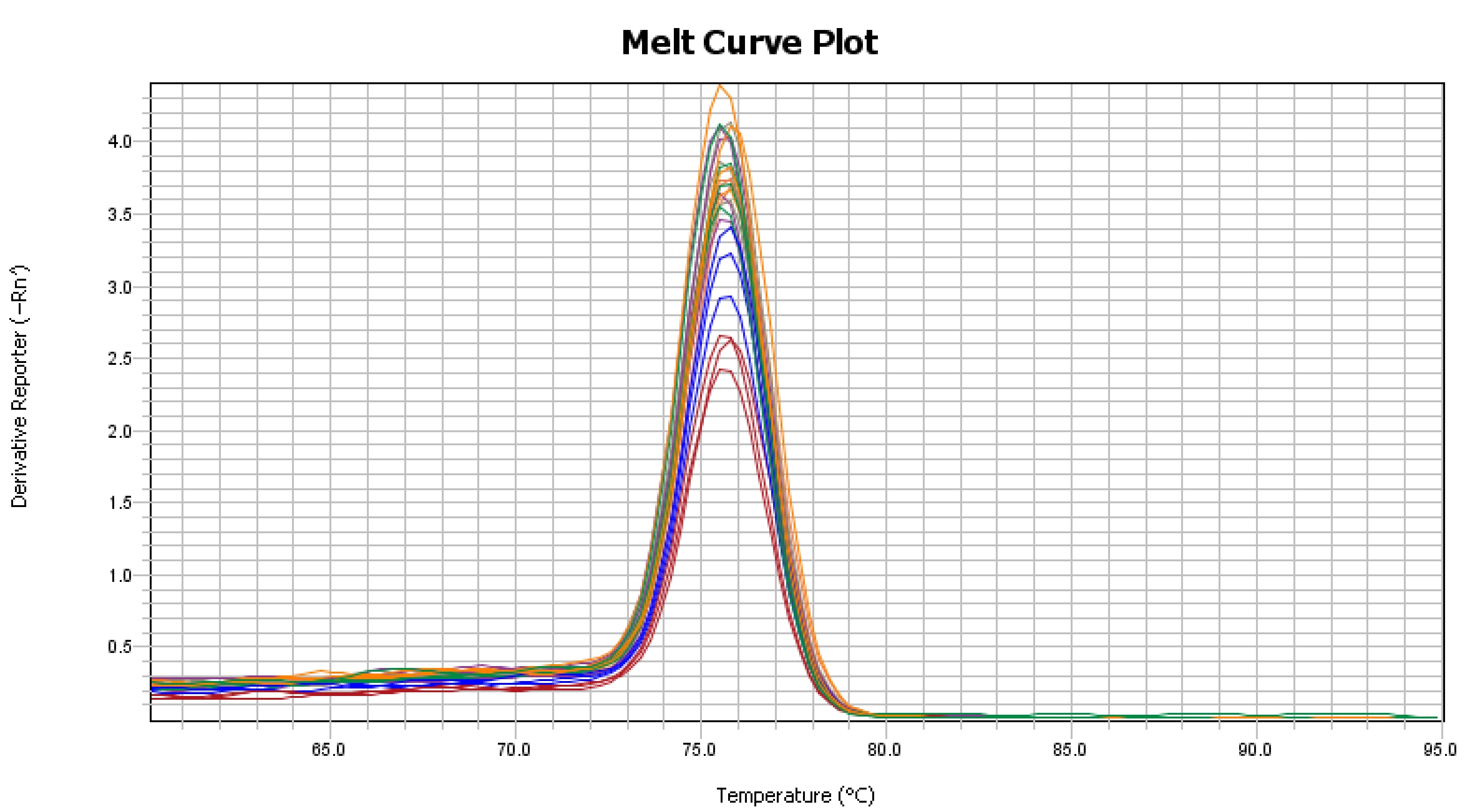

Supplement: Supplementary file 1 [file ijms-23-14464-s001.zip › Melt curve/Senescence associated genes/SGR.jpg]

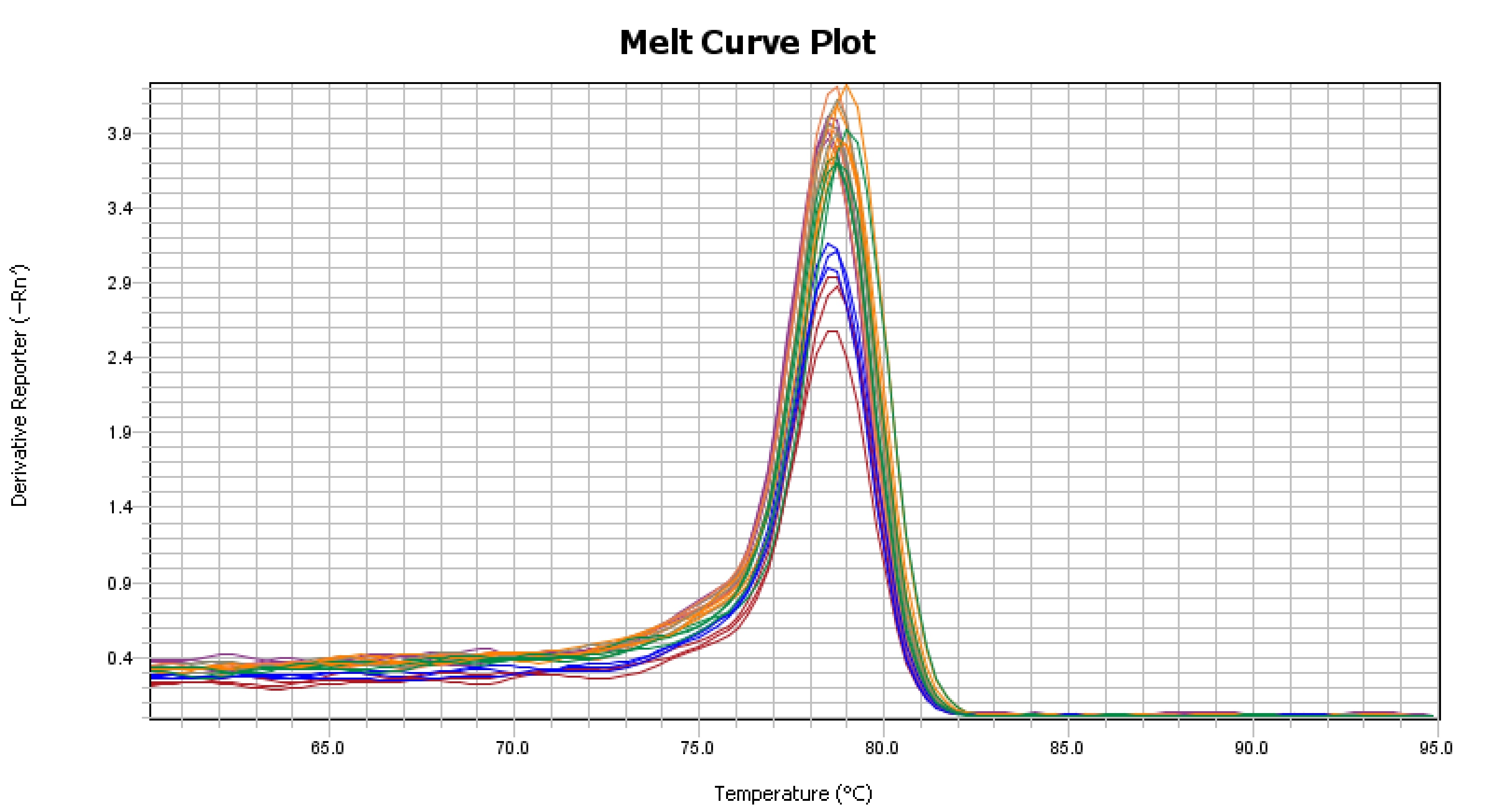

Supplement: Supplementary file 1 [file ijms-23-14464-s001.zip › Melt curve/Senescence associated genes/SODA1.jpg]

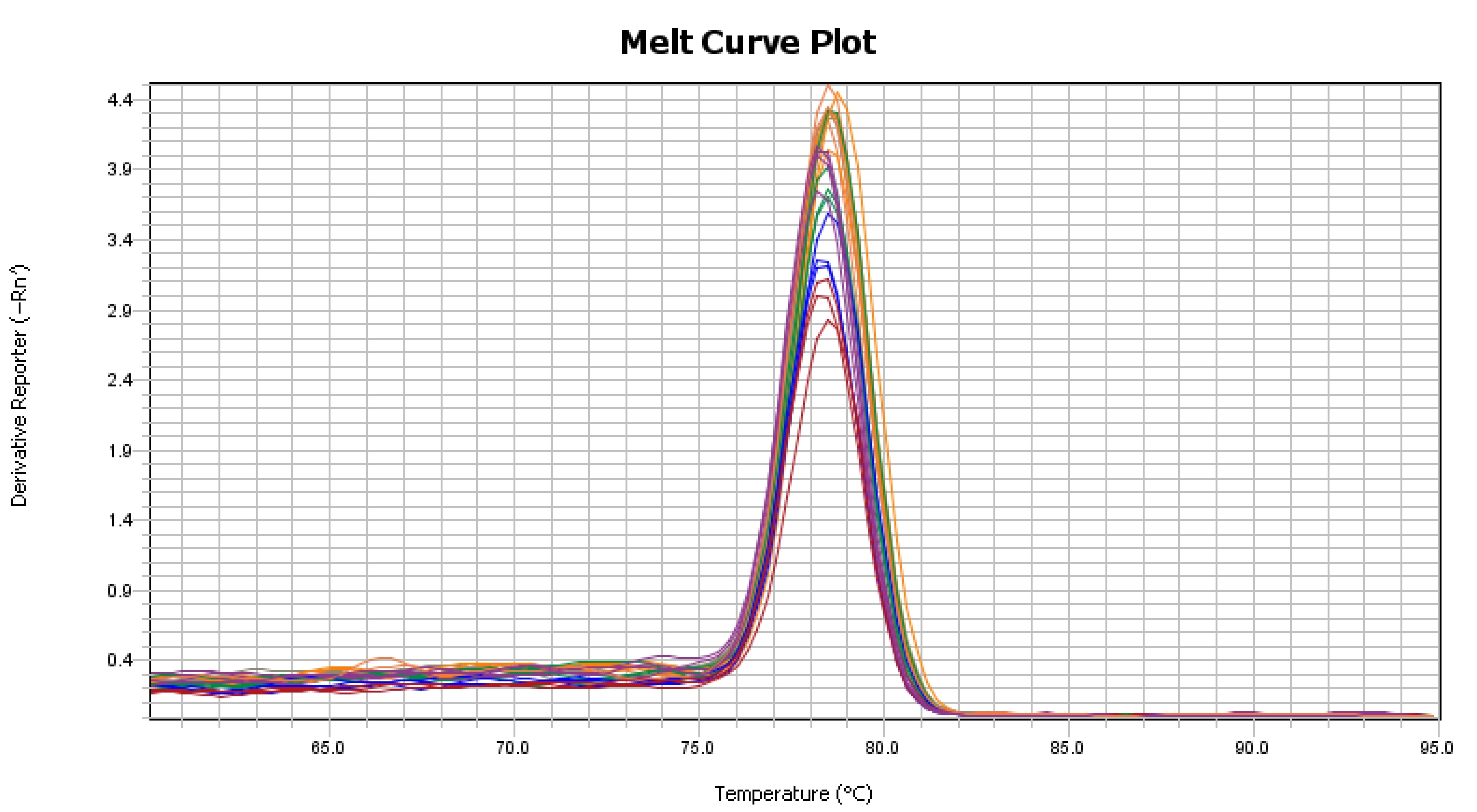

Supplement: Supplementary file 1 [file ijms-23-14464-s001.zip › Melt curve/Senescence associated genes/SODB.jpg]
